# HIV_GKO_: A Tool to Assess HIV-1 Latency Reversal Agents in Human Primary CD4^+^ T Cells

**DOI:** 10.21769/BioProtoc.3050

**Published:** 2018-10-20

**Authors:** Emilie Battivelli, Eric Verdin

**Affiliations:** 1 Gladstone Institute of Virology and Immunology, Gladstone Institutes, San Francisco, CA, USA; 2 Department of Medicine, University of California San Francisco, San Francisco, CA, USA; 3 Buck Institute for Research on Aging, Novato, CA, USA

**Keywords:** HIV-1 latency, Latency reversal, Latency reversal agents, Reservoirs, Human cells, Dual-fluorescence reporter, HIV_GKO_, Flow cytometry

## Abstract

While able to suppress HIV replication in HIV infected individuals, combination antiretroviral therapy (ART) fails to eliminate viral latent reservoir, which consists in integrated transcriptional silenced HIV provirus. So far, identification of latently-infected cells has relied on activating cells to induce expression of HIV proteins which can then be detected. Unfortunately, this activation significantly changed the cell phenotype. We developed a novel HIV reporter, named HIV_GKO_, that allows the purification of latently-infected cells in absence of reactivation. Indeed, latent cells can be identified by expression of the EF1a-driven mKO2 and lack of expression of the LTR-driven csGFP. This protocol can be used to study the effectiveness of LRAs (Latency Reversal Agents) in reactivating latent HIV in primary cells.

## [Background]

The new version of dual-labeled virus (HIV_GKO_), contains a codon-switched eGFP (csGFP) under the control of the HIV-1 promoter in the 5’ LTR and a distinct, unrelated fluorescent protein mkO2 under the control of the cellular elongation factor one alpha promoter (EF1α). It is important to use unrelated fluorescent proteins in those reporters due to recombination issues when using fluorescent proteins genetically related. Productively infected cells are thus mostly csGFP^+^ mKO2^+^ (some might only be GFP^+^), while latently infected cells are csGFP^−^ mKO2^+^. Flow cytometers such as the sorter Ariall allows the purification of pure infected population (productive, latent and/or uninfected), while the analyzer LSRII allows for the assessment of the transcriptional activation of the HIV_GKO_ LTR within a short time frame post-infection.

### Materials and Reagents

- Production of HIV_GKO_ in HEK293T cells
  1. 182 cm^2^ tissue culture flask (VWR, catalog number: 10062-864)
  2. Tips
    0.1-10 μl (Fisher Scientific, Fisherbrand^™^, catalog number: 02-681-440)
    1-200 μl (Fisher Scientific, Fisherbrand^™^, catalog number: 02-707-502)
    101-1,000 μl (Fisher Scientific, Fisherbrand^™^, catalog number: 02-707-509)
  3. Pipettes
    2 ml aspirating pipettes (VWR, catalog number: 414004-265)
    5 ml (VWR, catalog number: 89130-896)
    10 ml (VWR, catalog number: 89130-898)
    25 ml (VWR, catalog number: 89130-900)
  4. 15 ml conical tube (VWR, catalog number: 89039-666)
  5. 1.5 ml Eppendorf tubes (Fisher Scientific, catalog number: 05-408-129)
  6. 50 ml conical tube (VWR, catalog number: 89039-658)
  7. UltraClear Centrifuge Tubes 25 × 89 mm (Beckman Coulter, catalog number: 344058)
  8. 50 ml centrifuge tube filtration (VWR, catalog number: 89220-710)
  9. HEK293T cells (ATCC, catalog number: CRL-3216)
  10. Plasmids:
    - HIV_GKO_ (Battivelli et al., 2018)
    - HIV dual-tropic envelope (pSVIII-92HT593.1) (NIH AIDS Reagent Program, catalog number: 3077)
  11. DMEM (Corning, catalog number: 10-013-CVR)
  12. RPMI (Corning, catalog number: 10-040-CVR)
  13. Fetal bovine serum (FBS) (Gemini Bio-Products, BenchMark^™^, catalog number: 100-106)
  14. 100× penicillin/streptomycin (Corning, catalog number: 30-002-CI)
  15. 1× PBS (Corning, catalog number: 21-031-CVR)
  16. Trypsin-EDTA (Corning, catalog number: 25-053-CI)
  17. Cell culture water (Corning, catalog number: 25-055-CV)
  18. Chloroquine diphosphate salt (Sigma-Aldrich, catalog number: C6628)
  19. HEPES (Sigma-Aldrich, catalog number: H3375)
  20. Potassium chloride (KCI) (Sigma-Aldrich, catalog number: P9541)
  21. Dextrose (Fisher Scientific, catalog number: BP350-1)
  22. Sodium chloride (NaCI) (Sigma-Aldrich, catalog number: S3014)
  23. Sodium phosphate dibasic (Na_2_HPO_4_) (Fisher Scientific, catalog number: BP332-500)
  24. Calcium chloride (CaCl_2_) (Sigma-Aldrich, catalog number: C1016)
  25. Nuclease-free H_2_O (Thermo Fisher Scientific, Invitrogen^™^, catalog number: AM9937)
  26. FlaQ Assay reagents (Gesner et al., 2014)
  27. 25 mM chloroquine (see Recipes)
  28. 2× HBSS buffer (see Recipes)
  29. 2 M CaCl_2_ (see Recipes)
- Isolation of human primary CD4^+^ T cells
  1. 182 cm^2^ tissue culture flask (VWR, catalog number: 10062-864)
  2. Tips
    0.1-10 μl (Fisher Scientific, Fisherbrand^™^, catalog number: 02-681-440)
    1-200 μl (Fisher Scientific, Fisherbrand^™^, catalog number: 02-707-502)
    101-1,000 μl (Fisher Scientific, Fisherbrand^™^, catalog number: 02-707-509)
  3. Pipettes
    2 ml aspirating pipettes (VWR, catalog number: 414004-265)
    5 ml (VWR, catalog number: 89130-896)
    10 ml (VWR, catalog number: 89130-898)
    25 ml (VWR, catalog number: 89130-900)
  4. 50 ml conical tube (VWR, catalog number: 89039-658)
  5. Blood or LRC
  6. RosetteSep^™^ Human CD4^+^ T cell enrichment cocktail (STEMCELL Technologies, catalog number: 15062)
  7. Histopaque 1077 (Sigma-Aldrich, catalog number: 10771-500ML)
  8. RPMI (Corning, catalog number: 10-040-CVR)
  9. Fetal bovine serum (FBS) (Gemini Bio-Products, BenchMark^™^, catalog number: 100-106)
  10. 100× penicillin/streptomycin (Corning, catalog number: 30-002-CI)
  11. 1× PBS (Corning, catalog number: 21-031-CVR)
  12. Ammonium chloride (NH_4_Cl) (Sigma-Aldrich, catalog number: A9434-500G)
  13. Potassium bicarbonate (KHCO_3_) (Sigma-Aldrich, catalog number: 237205-100G)
  14. Ethylenediaminetetraacetic acid disodium salt dihydrate (Na_2_EDTA) (Sigma-Aldrich, catalog number: E6635-100G)
  15. Recombinant human Interleukin-2 (R&D Systems, catalog number: 202-IL-010)
  16. AKC lysis buffer (see Recipes)
- Infection of human primary CD4^+^ T-cells with HIV_GKO_
  1. 182 cm^2^ tissue culture flask (VWR, catalog number: 10062-864)
  2. Tips
    0.1-10 μl (Fisher Scientific, Fisherbrand^™^, catalog number: 02-681-440)
    1-200 μl (Fisher Scientific, Fisherbrand^™^, catalog number: 02-707-502)
    101-1,000 μl (Fisher Scientific, Fisherbrand^™^, catalog number: 02-707-509)
  3. Pipettes
    2 ml aspirating pipettes (VWR, catalog number: 414004-265)
    5 ml (VWR, catalog number: 89130-896)
    10 ml (VWR, catalog number: 89130-898)
    25 ml (VWR, catalog number: 89130-900)
  4. 50 ml conical tube (VWR, catalog number: 89039-658)
  5. 15 ml conical tube (VWR, catalog number: 89039-666)
  6. 96-well plate V-bottom (Thermo Fisher Scientific, Nunc^™^, catalog number: 249570) and lids (Thermo Fisher Scientific, catalog number: 263339)
  7. Pipetting reservoirs (VWR, catalog number: 89094-662)
  8. Isolated CD4^+^ T cells
  9. Viral stock
  10. RPMI (Corning, catalog number: 10-040-CVR)
  11. Fetal bovine serum (FBS) (Gemini Bio-Products, BenchMark^™^, catalog number: 100-106)
  12. 100× penicillin/streptomycin (Corning, catalog number: 30-002-CI)
  13. Recombinant human Interleukin-2 (R&D Systems, catalog number: 202-IL-010)
  14. Dynabeads human T-activator CD3/CD28 (Thermo Fisher Scientific, catalog number: 111.32D)
- Sorting cells
  1. Tips
    0.1-10 μl (Fisher Scientific, Fisherbrand^™^, catalog number: 02-681-440)
    1-200 μl (Fisher Scientific, Fisherbrand^™^, catalog number: 02-707-502)
    101-1,000 μl (Fisher Scientific, Fisherbrand^™^, catalog number: 02-707-509)
  2. Pipettes
    2 ml aspirating pipettes (VWR, catalog number: 414004-265)
    5 ml (VWR, catalog number: 89130-896)
    10 ml (VWR, catalog number: 89130-898)
    25 ml (VWR, catalog number: 89130-900)
  3. 50 ml conical tube (VWR, catalog number: 89220-710)
  4. 15 ml conical tube (VWR, catalog number: 89039-666)
  5. 96-well plate V-bottom (Thermo Fisher Scientific, Nunc^™^, catalog number: 249570) and lids (Thermo Fisher Scientific, catalog number: 263339)
  6. Falcon round-bottom 5 ml tubes with 35 μm cell strainer cap (Corning, catalog number: 352235)
  7. RPMI (Corning, catalog number: 10-040-CVR)
  8. Fetal bovine serum (FBS) (Gemini Bio-Products, BenchMark^™^, catalog number: 100-106)
  9. 100× penicillin/streptomycin (Corning, catalog number: 30-002-CI)
  10. Recombinant human Interleukin-2 (R&D Systems, catalog number: 202-IL-010)
  11. 1× PBS (Corning, catalog number: 21-031-CVR)
  12. Ethylenediaminetetraacetic acid disodium salt dihydrate (Na_2_EDTA) (Sigma-Aldrich, catalog number: E6635-100G)
  13. Sodium azide (NaN_3_) (Sigma-Aldrich, catalog number: S2002)
  14. FACS buffer (see Recipes)
- Drug treatment
  1. Tips
    0.1-10 μl (Fisher Scientific, Fisherbrand^™^, catalog number: 02-681-440)
    1-200 μl (Fisher Scientific, Fisherbrand^™^, catalog number: 02-707-502)
    101-1,000 μl (Fisher Scientific, Fisherbrand^™^, catalog number: 02-707-509)
  2. RPMI (Corning, catalog number: 10-040-CVR)
  3. Fetal bovine serum (FBS) (Gemini Bio-Products, BenchMark^™^, catalog number: 100-106)
  4. 100× penicillin/streptomycin (Corning, catalog number: 30-002-CI)
  5. Recombinant human Interleukin-2 (R&D Systems, catalog number: 202-IL-010)
  6. LRAs
- Analysis of LRAs efficacy by flow cytometry.
  1. RPMI (Corning, catalog number: 10-040-CVR)
  2. Fetal bovine serum (FBS) (Gemini Bio-Products, BenchMark^™^, catalog number: 100-106)
  3. 100× penicillin/streptomycin (Corning, catalog number: 30-002-CI)
  4. Recombinant human Interleukin-2 (R&D Systems, catalog number: 202-IL-010)
  5. 1× PBS (Corning, catalog number: 21-031-CVR)
  6. Ethylenediaminetetraacetic acid disodium salt dihydrate (Na_2_EDTA) (Sigma-Aldrich, catalog number: E6635-100G)
  7. Sodium azide (NaN_3_) (Sigma-Aldrich, catalog number: S2002)
  8. LIVE/DEAD Violet Fixable Dead Cell Stain Kit (Thermo Fisher Scientific, catalog number: L34963) (small kit)
  9. FACS buffer (see Recipes)

### Equipment

1. Pipetman
2. Flnnpipette^™^ F2 multichannel pipettes
  5-50 μl (Thermo Fisher Scientific, catalog number: 4662050)
  30-300 μl (Thermo Fisher Scientific, catalog number: 4662070)
3. Pipette-Aids
4. Tabletop centrifuge for Eppendorf tubes (Eppendorf, model: 5415D)
5. Vortex (VWR, catalog number: 10153-838)
6. Tabletop centrifuge for 96-well plates, Eppendorf, 15 ml and 50 ml tubes; used for spin infection (Beckman Coulter, model: Allegra X-14R)
7. High Speed Ultracentrifuge (Beckman Coulter, model: Optima L-60) with SW 28 Ti Swinging-Bucket rotor (Beckman Coulter, model: SW28 Ti)
8. 5% CO_2_ tissue culture incubator, 37 °C (Thermo Fisher Scientific, model: Forma^™^ Steri-Cult^™^ CO_2_ Incubators, catalog number: 3307)
9. DynaMag^™^-5 Magnet (Thermo Fisher Scientific, catalog number: 12303D)
10. FACS Ariall (BD Biosciences, San Jose, CA)
11. FACS LSRII (BD Biosciences, San Jose, CA)
12. Hemacytometer (Hausser Scientific, catalog number: 3200) or automated cell counter (ORLFO Technologies, catalog number: MXZ001) with cassettes type M (ORLFO Technologies, catalog number: MXC001)
13. Biosafety cabinet ‘Level 2’
14. Ice bucket (VWR, catalog number: 10146-298)

### Software

1. FlowJo 10 or other versions (Tree Star)

### Procedure

**Figure 1.**
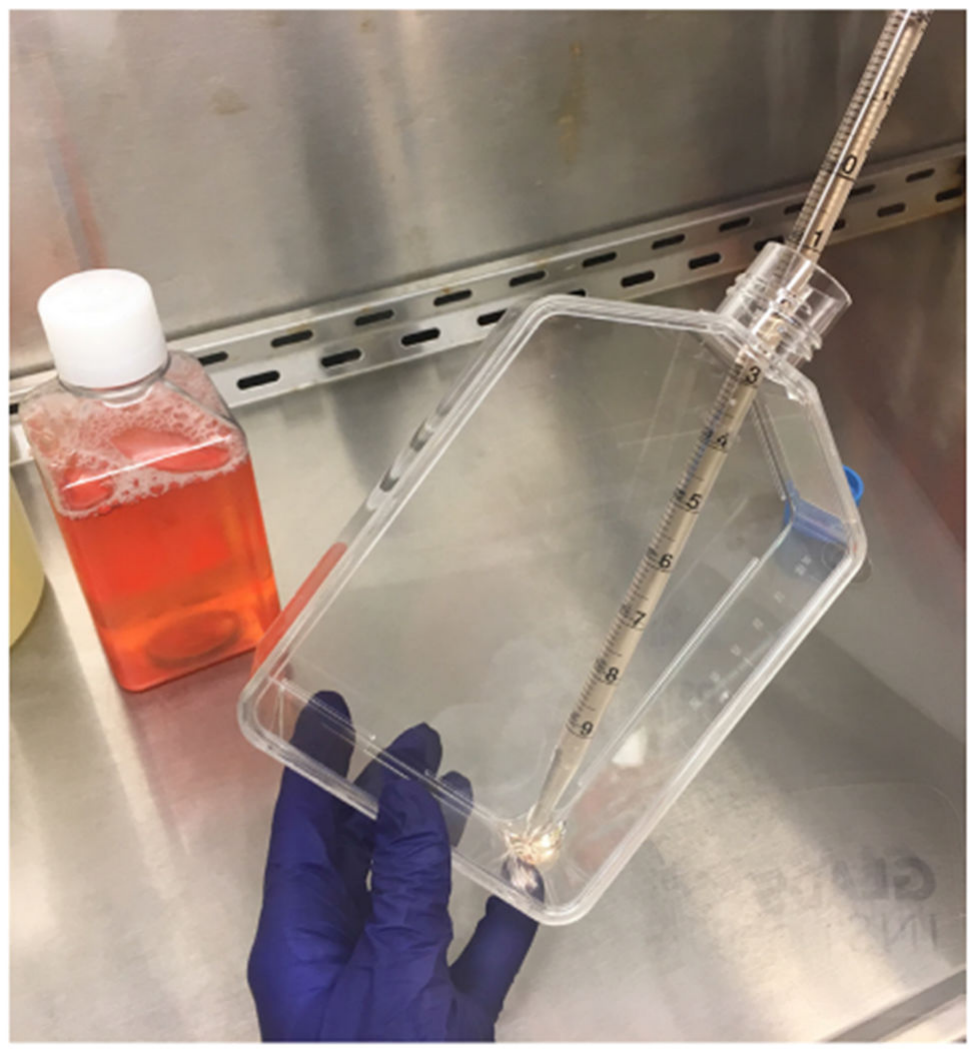
Adding media to cells without detaching cells. Steps A2b and A2k require to add fresh media with and without chloroquine, respectively. First, aspirate media, and then add fresh media directly at the bottom of the flask to prevent detaching the cells. Once the media is in the bottom of the flask, slowly swirl the flask to distribute the solution evenly and place the flask back into the incubator.

**Figure 2.**
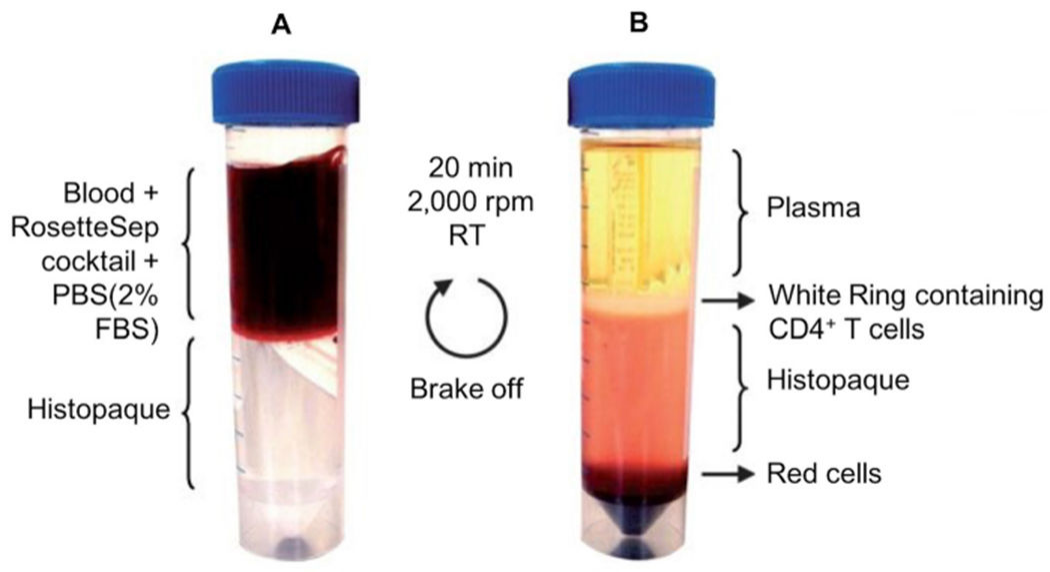
Isolation of human primary CD4T^+^ cells by histopaque density gradient. A. Slowly layer the blood (containing the RosetteSep Cocktail and diluted to half with PBS + 2% FBS) on top of histopaque density gradient. Centrifuge for 20 min at 800 *× g*, RT, with brake off. B. CD4^+^ T cells are contained in the white ring, below the plasma phase, but above the histopaque and red cell phases. Carefully pipet out the ring.

**Figure 3.**
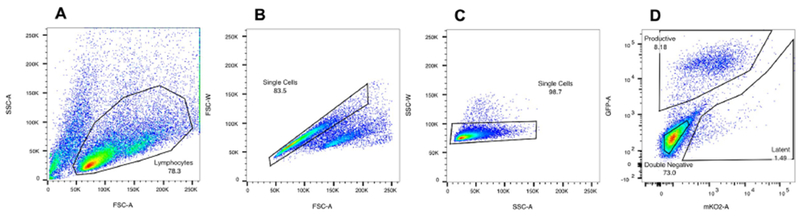
Gating strategy to sort out uninfected, productively and latently infected cells. A. Set the gate on live cells. Cell viability is monitored by forward (FSC-Area) and side scatter (SSC-Area) analysis. B and C. Gate successively on singlets FSC-Area vs. FSC-Width, and SSC-Area vs. SSC-Width. D. Set the gate on GFP/FITC-Area^+^ to sort productively infected cells, or on GFP/FITC-Area^−^ vs. mKO2/PE-Area^+^ to sort latently infected cells, or to GFP/FITC-Area^−^ vs. mKO2/PE-Area^−^ to sort uninfected cells. Run briefly each sorted sample when the sort is over to check purity (usually > 90%).

**Figure 4.**
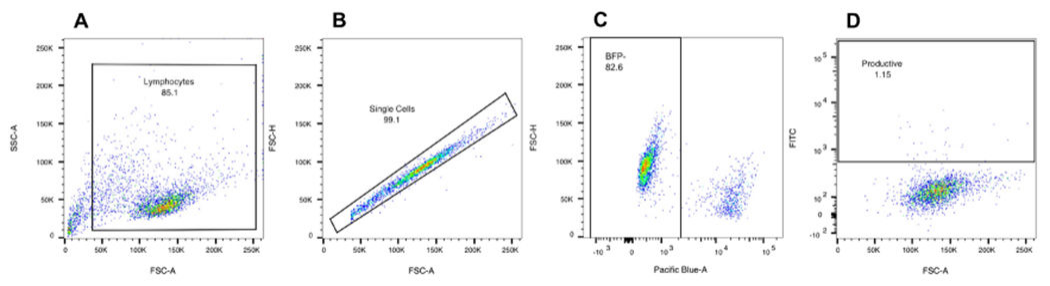
Gating strategy to analyze LRAs efficacy on the reversal of HIV-1 latency. A. Set the gate cells (avoid the left bottom corner where it is mainly debris) based on size (FSC-Area) and granularity (SSC-Area) analysis. B. Gate on singlets (FSC-Area vs. FSC-Height). C. Gate on live cells (Live) (Pacific Blue-Area vs. FSC-Height). D. Quantify the number of GFP^+^ cells which is the number of productively infected cells (GFP/FITC-Area^+^ vs. FSC-Area). Deduce the % of GFP^+^ cells quantified in the control sample to obtain the number of latently reactivated cells by the LRA tested. Each LRA is tested at least on 3 independent HIV_GKO_ infected C donors.

- Production of HIV_GKO_ viral particles in HEK293T cells
  Note: HIV_GKO_ particles are produced after calcium phosphate transfection in HEK293T cells.
  1. HEK293T cell culture
    - HEK293T cells are cultured in DMEM medium (supplemented with 10% FBS, 1% penicillin/streptomycin) in 182 cm^2^ tissue culture flasks in 20 ml medium.
    - For maintenance of the HEK293T culture, when approaching confluence (~80%) cells, aspirate media, wash once with PBS, then trypsinize (0.05% trypsin) and plate cells after 1/12 dilution in complete DMEM, in 182 cm^2^ tissue culture flasks. HEK293T cells are split every 3 days.
    - One day before transfection, plate approximately 4 × 10^6^ of HEK293T cells per 182 cm^2^ tissue culture flasks in 18 ml of complete DMEM.
    Note: After thawing frozen cell vials, HEK293T cells are cultured for at least one week before transfecting them for virus production. In order to maximize viral particle production, HEK293T cells are never kept more than 4 weeks in culture. HEK293T cells should never be grown to 100% confluency as they lose their ability to be transfected.
  2. Calcium phosphate transfection
    Note: Protocol below describes the transfection of cells plated in one 182 cm^2^ tissue culture flask. To utilize the maximum space available in the ultracentrifuge, transfect about 15 flasks.
    - Remove all medium from the flask.
    - Add 17 ml of fresh DMEM medium (supplemented with 10% FBS, 1 % penicillin/streptomycin) containing a final concentration of 25 μM of chloroquine (do not add on cells but, at the bottom of the flask–Figure 1), slowly swirl the flask to distribute the solution evenly and, incubate for 30-60 min before transfection.
    - Prepare DNA mix in nuclease-free H_2_O in a 15 ml conical tube (45 μg of HIV_GKO_ and 25 μg of HIV-1 dual-tropic envelope [pSVIII-92HT593.1]).
    - Add nuclease-free H_2_O to the DNA mixture for a final volume of 1.5 ml.
    - Add 1.75 ml of 2× HBSS buffer.
    - Vortex mixture at a medium-high setting while slowly adding (dropwise) 220 μl of 2 M CaCl_2_ to the diluted DNA, and keep vortexing for about 45 sec.
    - Incubate at room temperature for 10-30 min before adding the prepared DNA solution to the bottom of the flask.
    - Slowly swirl the flask to distribute the solution evenly.
    - Culture for 6-8 h at 37 °C.
    - Remove supernatant.
    - Add 17 ml of DMEM (supplemented with 10% FBS, 1% penicillin/streptomycin) medium (add at the bottom of the flask, as shown in Figure 1, to avoid detaching cells).
    - Incubate for 48 h at 37 °C to allow viral production.
    - Collect the supernatant into a 50 ml Falcon tube.
    - Centrifuge for 20 min at 800 × *g* at RT or 4 °C.
    - Filter supernatant through a 50 ml centrifuge tube filtration.
    - Transfer filtered supernatant into UltraClear Centrifuge Tubes (To avoid the collapse of the tube, make sure it is filled with at least 34 ml–add media or PBS to viral supernatant if necessary to reach that volume).
    - Spin viral supernatant in the ultracentrifuge for 2 h at 100,000 *× g* at 4 °C.
    - Pour out supernatant, dry as much as you can the inside of the tube and resuspend the pellet with 50 μl of cold RPMI (supplemented with 10% FBS, 1% penicillin/streptomycin) medium or FBS, make aliquots in 1.5 ml Eppendorf tubes and freeze at −80 °C.
      Notes:
      - When scaling up the viral production, you should be able to see a pellet. Also, scale up the volume used to resuspend the pellet. Do not make air bubbles when resuspending the pellet and, pipet up and down at least 50 times.
      - Concentrated viral supernatant can also be titered and used fresh. However, even though the p24 content will not change, fresh versus frozen virus will give different infection outcomes, which will require different viral input to start with (described in Step C2).
    - Thaw one aliquot, make viral dilutions up to 10,000 to 1 million and, titer virus for p24 content using the FlaQ assay protocol (Gesner et al., 2014).
      Note: In addition of tittering p24 content of the virus with the FlaQ assay, I would recommend directly titering the infection rate of your viral stock on activated cells using 4 or 5 viral dilutions, before proceeding with big experiments. You want to avoid total infection greater than 15%.
- Isolation of human primary CD4^+^ T cells
  1. Human primary CD4^+^ T cells culture
    - Human primary CD4^+^ T cells are cultured in RPMI medium (supplemented with 10% FBS, 1% penicillin/streptomycin) + 20 U/ml of IL-2 in tissue culture flasks or plates at a concentration of 5 × 10^6^/ml, in a CO_2_ incubator at 37 °C.
    - Half of the medium is replaced with fresh RPMI medium (supplemented with 10% FBS, 1% penicillin/streptomycin) + 20 U/ml of IL-2 every other day.
  2. Isolation of human primary CD4^+^ T cells
    Note: This protocol describes CD4^+^ T cells isolation using the RosetteSep Human CD4^+^ T Cell Enrichment Cocktail. Any other CD4^+^ T cells isolation kit can be used, but the protocol might slightly differ. Always follow the manufacturer’s protocol.
    - Order fresh blood or LRC in advance to have it delivered on the day of the experiment.
      Note: One blood/LRC corresponds to one donor. Three donors should be tested at least, in two different experiments at least.
    - Transfer blood into a 50 ml Falcon tube (if using LRC, cut both extremities of the chamber with clean scissors and let the blood drops into the tube).
    - Add 1,800 μl of RosetteSep Cocktail to sample, incubate at RT for 20 min, and mix sample by swirling the tube every 5 min.
    - In a new 50 ml Falcon tube, add 10 ml of Histopaque-1077.
    - Dilute sample with equal volume of PBS containing 2% FBS.
    - Slowly and carefully layer diluted sample on density gradient medium to minimize their mixing.
    - Centrifuge sample for 20 min at 800 *× g*, RT, with **brake off**.
    - CD4^+^ T cells are contained in the white ring, below the plasma phase (Figure 2). Pipet the ring and transfer it to a new 50 ml Falcon tube.
    - Wash cells by filling up the tube with PBS containing 2% FBS.
    - Centrifuge for 3 min at 800 *× g* and discard supernatant.
    - If pellet appears red, resuspend pellet with 15 ml of **cold** AKC lysis buffer (see Recipes), incubate for 2 min at RT.
    - Fill the tube up to 50 ml with PBS containing 2% fetal bovine serum, centrifuge for 3 min at 800 *× g*, and get rid of the supernatant.
    - If the pellet is still red, repeat Steps B2k-B2l, if not, proceed to Step B2n.
    - Resuspend pellet (CD4^+^ T cells) with warm, fresh RPMI medium (supplemented with 10% FBS, 1% penicillin/streptomycin) + 20 U/ml of IL-2.
    - Count cells using an automated cell counter or a hemacytometer.
    - Culture cells at a concentration of 5 × 10^6^/ml (250 million cells are cultured into 185 cm^2^ [flat]).
      Note: Isolated CD4^+^ T cells are mainly resting and can be kept in culture as such for several days. Medium can be changed every 4 to 5 days.
- Infection of human primary CD4^+^ T-cells with HIV_GKO_
  Note: This protocol describes the activation of 250 million CD4^+^ T cells using Dynabeads Human T-Activator CD3/CD28. Any other human CD4^+^ T cells activators kit can be used, but the protocol might slightly differ. Always follow the manufacturer’s protocol.
  1. Activation of human primary CD4^+^ T cells
    - Vortex the Dynabeads Human T-Activator CD3/CD28 in the vial.
    - Transfer 3.125 ml of Dynabeads (1 bead for 2 cells, which is half of the manufacturer’s protocol) to a 15 ml tube.
    - Place the tube on a magnet for 1 min and discard the supernatant.
    - Remove the tube from the magnet and resuspend the washed Dynabeads with 10 ml of the CD4^+^ T cells to activate (at a concentration of 5 × 10^6^/ml), and add those 10 ml to the rest of the CD4^+^ T cells to activate. Transfer the whole suspension to an appropriate vessel (5 millions of activated cells can be cultured in one well of a 24-well plate).
    - Culture in a CO_2_ incubator at 37 °C for three days as such (you should see cells aggregates due to activation).
  2. Infection of human primary CD4^+^ T cells with HIV_GKO_
    *Note: The spin-infection of 1 million activated CD4*^+^ *T cells requires 30 μl of a 10,000 ng p24^Gag^/ml viral dilution to reach a total infection rate of 9%-12% (to keep the ratios of latent versus productive infections consistent, avoid infection rates greater than 15%). However, the total infection rate (productive* + *latent infections) might slightly change according to fresh versus frozen stocks, viral stocks themselves and donors. Thus, in addition of titering the virus with the FlaQ assay, I would recommend directly titering your viral stock on activated cells using 4 or 5 viral dilutions, before proceeding with big experiments. Using frozen viral stocks requires the use of bigger amount of virus to reach 9%-12% infection rate.*
    - Three days post-activation, mix cells and beads and, transfer cells to 15 ml tubes.
    - Place the tube on a magnet for 1 min and transfer the supernatant into a new 50 ml tube.
    - Centrifuge cells for 3 min at RT at 800 *× g*.
    - Discard supernatant and resuspend the cell pellet of 250 million cells with media containing the viral preparation at 10,000 ng p24^Gag^/ml.
    - Transfer resuspended cells to a pipetting reservoir and distribute 30 μl of the cells/virus suspension per one well of a 96 well V-bottom plate using a multi-channel pipetman.
    - Centrifuge (= spin-infection) the plate for 2 h, at 800 *× g*, 32 °C.
    - Add 100 μl of fresh RPMI medium (supplemented with 10% FBS, 1% penicillin/streptomycin) + 20 U/ml of IL-2 in each well and pool cells back together. Resuspend cells in a final volume of 100 ml of fresh RPMI medium (supplemented with 10% FBS, 1% penicillin/streptomycin) + 20 U/ml of IL-2 and, transfer into a 182 cm^2^ flask.
      Note: Viral solution stays in, but it is possible to wash it away after spin-infection.
    - Culture as such for 4 to 5 days in a CO_2_ incubator at 37 °C.
    - Replace half of the medium by centrifuging down cells, with fresh RPMI medium (supplemented with 10% FBS, 1% penicillin/streptomycin) + 20 U/ml of IL-2 every other day.
- Sorting cells
  1. Five days post-infection, collect and transfer cells to 50 ml tubes.
  2. Centrifuge cells for 3 min at RT at 800 *× g*.
  3. Discard supernatant and resuspend cells in 2 ml of FACS buffer (see Recipes).
  4. Pipette resuspended cells through a 5 ml 35 μm cell strainer capped tube and place on ice.
  5. Prepare several 15 ml collection tubes with 2 ml of FBS and place on ice. Do not forget to label your tubes.
  6. Then proceed directly to cell sorting using the flow cytometer FACS Ariall (PE channel for mkO2, and FITC channel for GFP) (see Figure 3 for gating strategy).
    Note: The sort can take up to 10 h, thus sort cells at 4 °C. Use the 85 μm nozzle to prevent spontaneous reactivation of latently infected cells. When collecting populations into 15 ml tubes, you can only collect 2 different populations at once. Keep sorting the latent population at all time, and exchange tubes for uninfected and productively infected cells when you have reached 5 million cells per population. You should be able to collect 1.5-2.5 million latently infected cells depending on your infection rate.
  7. Spin down sorted cells for 3 min at RT, 800 *× g*.
  8. Resuspend cell populations with fresh RPMI medium (supplemented with 10% FBS, 1% penicillin/streptomycin) + 20 U/ml of IL-2 and, distribute equally into a 96-well V-bottom plate.
    Note: To test 5 LRAs, sorted populations should be divided equally into 5 wells. Given that each well contains 200 μl, the pellet should be resuspended in 1 ml final of RPMI media. Note that a few hundred thousand cells/well are enough to assay LRAs activity.
  9. Let cells rest overnight in a CO_2_ incubator at 37 °C.
- Drug treatment
  1. After 24 h incubation, prepare 2× LRAs dilutions (dilute drugs with complete RPMI).
  2. Remove 100 μl of medium from each well and add 100 μl of 2× concentrated LRAs dilutions.
  3. Incubate for 24 h in a CO_2_ incubator at 37 °C.
- Analysis of LRAs efficacy by flow cytometry
  1. Twenty-four hours later, remove all medium from wells, and wash with FACS buffer.
  2. Spin down cells for 3 min at RT, 800 *× g* and discard supernatant.
  3. Stain cells with live/dead violet marker (1/1,000 dilution of the marker in FACS buffer, 100 μl/well, incubate for 10-15 min on ice in the dark).
    Note: Live/dead violet marker is perfect since it does not overlap with FITC and PE channels, and thus no compensation is needed.
  4. Wash cells once with FACS buffer and, proceed directly to flow analysis (see Figure 4 for gating strategy).
    Notes:
    - Avoid fixing samples with PFA since it decreases fluorescence intensity.
    - For flow cytometry, Ariall sorter was used to run samples since fluorescence intensity is higher. However, other flow cytometers such as LSRII or Calibur are also suitable for these experiments as long as they have the right filters.

### Data analysis

1. Sorting Cells (Figure 3)
2. Analysis of LRAs efficacy by flow cytometry (Figure 4)

### Notes

The HIV_GKO_ construct has a defective envelop and requires the addition of exogenous envelop while producing viral particles. We use an HIV CXCR4 tropism envelop to target human primary CD4^+^ T cells. It is possible to pseudotype that construct with other envelops such as VSV-G or HIV CCR5 tropism envelops to target other cell types (Cavrois et al., 2006).

### Recipes

1. 25 mM chloroquine
  25 mM chloroquine in PBS
2. 2× HBSS
  50 mM HEPES
  10 mM KCl
  12 mM dextrose
  280 mM NaCl
  1.5 mM Na_2_HPO_4_
  Adjust pH to 7.1
  Note: The pH is crucial!
3. 2 M CaCl_2_
  2 M CaCl_2_ in nuclease-free H_2_O
4. AKC Lysis buffer
  850 ml H_2_O
  8.02 g (150 mM) of NH_4_Cl
  1 g (10 mM) of KHCO_3_
  37.2 mg (0.1 mM) of Na_2_EDTA
  Adjust pH to 7.2
  Add H_2_O to 1,000 ml, and store at 4 °C for months
5. FACS buffer
  2% FBS
  2 mM EDTA
  0.1% Sodium Azide
  PBS
